# Blood molybdenum level as a marker of cancer risk on BRCA1 carriers

**DOI:** 10.1186/s13053-024-00291-7

**Published:** 2024-09-19

**Authors:** Milena Matuszczak, Adam Kiljańczyk, Wojciech Marciniak, Róża Derkacz, Klaudia Stempa, Piotr Baszuk, Marta Bryśkiewicz, Cezary Cybulski, Tadeusz Dębniak, Gronwald Jacek, Tomasz Huzarski, Marcin Lener, Anna Jakubowska, Sandra Pietrzak, Marek Szwiec, Małgorzata Stawicka-Niełacna, Dariusz Godlewski, Artur Prusaczyk, Andrzej Jasiewicz, Tomasz Kluz, Joanna Tomiczek-Szwiec, Ewa Kilar-Kobierzycka, Monika Siołek, Renata Posmyk, Joanna Jarkiewicz-Tretyn, Rodney Scott, Steven Narod, Jan Lubiński

**Affiliations:** 1 Department of Genetics and Pathology, International Hereditary Cancer Center, Pomeranian Medical University, ul. Unii Lubelskiej 1, Szczecin, 71-252 Poland; 2 Read-Gene, Dobra (Szczecińska), ul. Alabastrowa 8, Grzepnica, 72-003 Poland; 3 Department of Clinical Genetics and Pathology, University of Zielona Góra, ul. Zyty 28, Zielona Góra, 65- 046 Poland; 4 Independent Laboratory of Molecular Biology and Genetic Diagnostics, Pomeranian Medical University in Szczecin, Szczecin, 70-204 Poland; 5 Department of Surgery and Oncology, University of Zielona Góra, Zyty 28, 65-046 Zielona, Góra, Poland; 6 OPEN, Kazimierza Wielkiego 24 St, Poznań, 61-863 Poland; 7 Medical and Diagnostic Center, Siedlce, 08-110 Poland; 8 Genetic Counseling Center, Subcarpatian Oncological Hospital, 18 Bielawskiego St, 36-200, Brzozów, Poland; 9 Department of Gynecology, Gynecology Oncology and Obstetrics, Institute of Medical Sciences, Medical College of Rzeszow University, Rejtana 16c, Rzeszow, 35-959 Poland; 10 Department of Histology, Department of Biology and Genetics, Faculty of Medicine, University of Opole, Opole, 45-040 Poland; 11 Department of Oncology, District Specialist Hospital, Leśna 27-29 St, 58-100, Świdnica, Poland; 12 Holycross Cancer Center, Artwińskiego 3 St, 25-734, Kielce, Poland; 13 Department of Clinical Genetics, Medical University in Bialystok, Bialystok, 15-089 Poland; 14 Non-Public Health Care Centre, Cancer Genetics Laboratory, Toruń, 87-100 Poland; 15 Medical Genetics, Hunter Medical Research Institute, Priority Research Centre for Cancer Research, Innovation and Translation, School of Biomedical Sciences and Pharmacy, Faculty of Health and Medicine, Pathology North, John Hunter Hospital, King and Auckland Streets, University of Newcastle, Newcastle, NSW 2300 Australia; 16 Women’s College Hospital, Women’s College Research Institute, University of Toronto, Toronto, ON M5G 1N8 Canada

**Keywords:** Molybdenum, breast cancer, Ovarian cancer, Cancer risk, BRCA1 carriers, Prospective cohor

## Abstract

**Objective:**

To investigate whether Molybdenum blood level is a marker of cancer risk on BRCA1 carriers.

**Methods:**

A prospective cohort study was conducted among 989 initially unaffected women with a BRCA1 mutation. Blood samples were collected to measure molybdenum levels, and participants were followed for an average of 7.5 years. Cox proportional hazards models were used to assess the association between blood molybdenum levels and cancer incidence, adjusting for potential confounders.

**Results:**

High blood molybdenum levels (> 0.70 µg/L) were significantly associated with an increased risk of developing ovarian cancer (HR = 5.55; 95%CI: 1.59–19.4; *p* = 0.007) and any cancer (HR = 1.74; 95%CI: 1.17–2.61; *p* = 0.007) but not breast cancer (HR = 1.46, CI = 0.91–2.33; *p* = 0.12). The cumulative incidence of ovarian cancer at ten years was 1.2% for the lowest molybdenum tertile, 4.2% for the middle tertile, and 8.7% for the highest tertile.

**Conclusion:**

Elevated blood molybdenum levels are associated with an increased risk of ovarian cancer on BRCA1 mutation carriers. Lowering molybdenum levels may potentially reduce cancer risk in this population, and high molybdenum levels could serve as a marker for considering preventive oophorectomy in BRCA1 carriers. Further research is warranted to confirm these findings and explore interventions targeting molybdenum levels as a preventive measure for ovarian cancer in BRCA1 mutation carriers.

## Introduction

Molybdenum is an essential trace element which is widely distributed in nature. It was named after the Greek word molybdos, meaning “similar to lead”.

Long-term occupational exposure to molybdenum has been associated with an elevated incidence of cardiovascular disease [1]. Molybdenum toxicity can disrupt the absorption of copper and elevate uric acid levels, resulting in symptoms resembling gout, such as joint pain and swelling. Additionally, it may cause abnormalities in the gastrointestinal tract, liver and kidneys [2].

According to the International Agency for Research on Cancer (IARC 2018), molybdenum trioxide (MoO3) is classified as possibly carcinogenic to humans (Group 2B) based on experimental animal studies [3]. Among those with occupational exposure to molybdenum, an increase in lung cancer risk was reported [4].

Women with an inherited *BRCA1* mutation have a lifetime risk of breast cancer up to 80% and a risk of ovarian cancer up to 40% [5]. The likelihood of developing cancer among mutation carriers is influenced by several modifying factors, including reproductive history (fertility, age at first birth and breastfeeding), exogenous hormones (oral contraceptives and hormone replacement therapy) and possibly lifestyle factors such as physical activity and diet. To date, little is known about the impact of exposure to low levels of molybdenum on women who have an inherited susceptibility to cancer.

Hence, we correlated blood levels of molybdenum and cancer risks on *BRCA1* mutation carriers in Poland.

## Materials and methods

The study subjects comprised 989 adult women, who received genetic counselling and testing between 2011 and 2017 at the Clinical Hospitals of Pomeranian Medical University in Szczecin, Poland, or at an associated hospitals or outpatient clinics. At the first study visit, a fasting blood sample was collected from each study participant to be used for genetic testing for *BRCA1* founder mutations. Within this cohort, 559 women had the c.5266dupC mutation, 250 had the c.181T/G mutation, 50 had c.4035delA, 18 had c.3700_3704delGTAAA, 14 had c.1687 C > T, 12 had c.5251 C > T, 9 had c.66_67delAG (or c.68_69delAG), and 9 had c.676delT; additionally, 68 women had other, less common mutations. For analysis, 10 mL of peripheral blood was collected into a vacutainer tube containing ethylenediaminetetraacetic acid (EDTA) from all study participants. All blood samples were collected between 8 a.m. and 2 p.m. and stored at − 80 ℃ until analysis. Participants were included in the study if a deleterious *BRCA1* variant was detected. Typically, these patients are offered the opportunity to participate in other clinical research studies. Medical charts were reviewed for age of diagnosis, oral contraceptive use (ever/never), hormone replacement therapy use (ever/never), smoking history (yes/no), oophorectomy (yes/no). The study was conducted in accordance with the Helsinki Declaration and with the consent of the Ethics Committee of Pomeranian Medical University in Szczecin under the number KB-0012/73/10 of 21 June 2010. All participants provided written informed consent.

### Measurement of blood molybdenum level

The blood samples were obtained from fasting individuals through venipuncture using the Vacutainer^®^ System (BD #368381, Plymouth, UK). Blood was carefully divided into new cryovials and then frozen at -80 °C until analysis.

The elemental composition of the samples was determined using the inductively coupled plasma mass spectrometry (ICP-MS) technique with the NexION 350D instrument (PerkinElmer, Norfolk, USA). The KED (Kinetic Energy Discrimination) mode was employed for element determination, and rhodium was used as an internal standard to compensate for instrument drift and matrix effects. Detailed information regarding the specific parameters of the NexION 350D instrument used in the measurements can be provided upon request. During analysis, the blood samples were diluted 40-fold with blank reagent (70 µl blood: 2730 µl buffer).

The blank reagent used consisted of high-purity water (> 18 MΩ), tetramethylammonium hydroxide (TMAH) (AlfaAesar, Kandel, Germany), Triton X-100 (PerkinElmer, Shelton, CT, USA), EDTA (Merck, Darmstadt, Germany), and ethyl alcohol (Merck, Darmstadt, Germany).

Calibration curve standards were prepared by diluting the stock solution of 1000 µg/ml Molybdenum Standard (PerkinElmer Pure Plus, Shelton, CT, USA) with the blank reagent. The calibration method used was matrix matched, and the correlation coefficients for calibration curve was always greater than 0.999.

The accuracy and precision of the measurements were evaluated using certified reference materials (CRM): ClinChek^®^ Plasmonorm Whole Blood Level 1 (Recipe, Munich, Germany) and Seronorm Whole Blood Level 2 (Sero, Norway). Technical details, plasma operating settings, and mass spectrometer acquisition parameters can be provided upon request. The testing laboratory participates in an independent external quality assessment scheme, QMEQAS (Quebec Multielement External Quality Assessment Scheme) organized by the Institut National de Santé Publique du Québec.

### Statistical analysis

All study participants were assigned to one of three categories (tertiles) depending on their blood molybdenum level. The cumulative risks of breast and ovarian cancer were calculated from the age at blood draw to the age of diagnosis of breast or ovarian cancer, death from another cause, or last follow up. For estimating the risk of ovarian cancer, women with oophorectomy prior to blood draw were excluded and subjects with oophorectomy in the follow-up period were censored at the time of oophorectomy. For the analysis of breast cancer risk, oophorectomy was included as a time-dependent variable. To estimate the ten-year cumulative risk of ovarian cancer, patients were followed from blood draw to date of preventive oophorectomy, ovarian cancer, ten years of follow up, last follow up or death from another cause. In order to estimate the hazard ratios (HRs) for cancer risk, univariable and multivariable Cox proportional hazards regression analyses were performed. In multivariable models, the following variables were taken into analysis: molybdenum level (tertile), year of birth, age at blood draw (< 40 years, 40–49.9 years ≥ 50 years), oral contraceptive use (yes/no), hormone replacement therapy use (yes/no), smoking history (current, former never), BMI (< 23.0 versus > 23.0). All statistical analyses were performed using SAS, version 9.4.

## Results

The research cohort consisted of 989 women with a *BRCA1* mutation who were unaffected at the time of inclusion in the study (date of blood draw). Over an average follow-up period of 7.5 years, 173 new cancers were reported (121 cases of breast cancer, 29 cases of ovarian cancer, and 23 cancers at other sites). Two study participants were diagnosed with both breast and ovarian cancer during the follow-up period. The characteristics of the study group are shown in Table 1.

**Table 1:** Group characteristics

	*N* = 989
Age at enrollment	
< 50 years ≥ 50 years	775 (78.36%) 214 (21.64%)
Smoking	
never ever missing data	720 (72.80%) 264 (26.70%) 5 (0.50%)
Hormonal therapy	
never ever missing data	720 (72.80%) 263 (26.59%) 6 (0.61%)
Oophorectomy	
no yes missing data	413 (41.89%) 573 (58.11%) 0 (0.00%)
Oral Contraceptive use	
never ever missing data	501 (50.51%) 481 (48.28%) 6 (1.21%)
Body Mass Index (kg/m2)	
< 18.5 18.5–24.9 25.0-29.9 ≥ 30.0 -missing data	56 (5.66%) 553 (55.92%) 237 (23.96%) 95 (9.61%) 48 (4.85%)
New cancer site	
breast ovarian bladder cervix colon kidney leukemia lung pancreas salivary gland sarcoma site unknown skin thyroid urothelial	121 (69.94%) 29 (16.75%) 2 (1.16%) 3 (1.73%) 2 (1.16%) 1 (0.58%) 2 (1.16%) 3 (1.73%) 1 (0.58%) 1 (0.58%) 1 (0.58%) 1 (1.16%) 1 (0.58%) 3 (1.73%) 1 (0.58%)

**Fig. 1:**
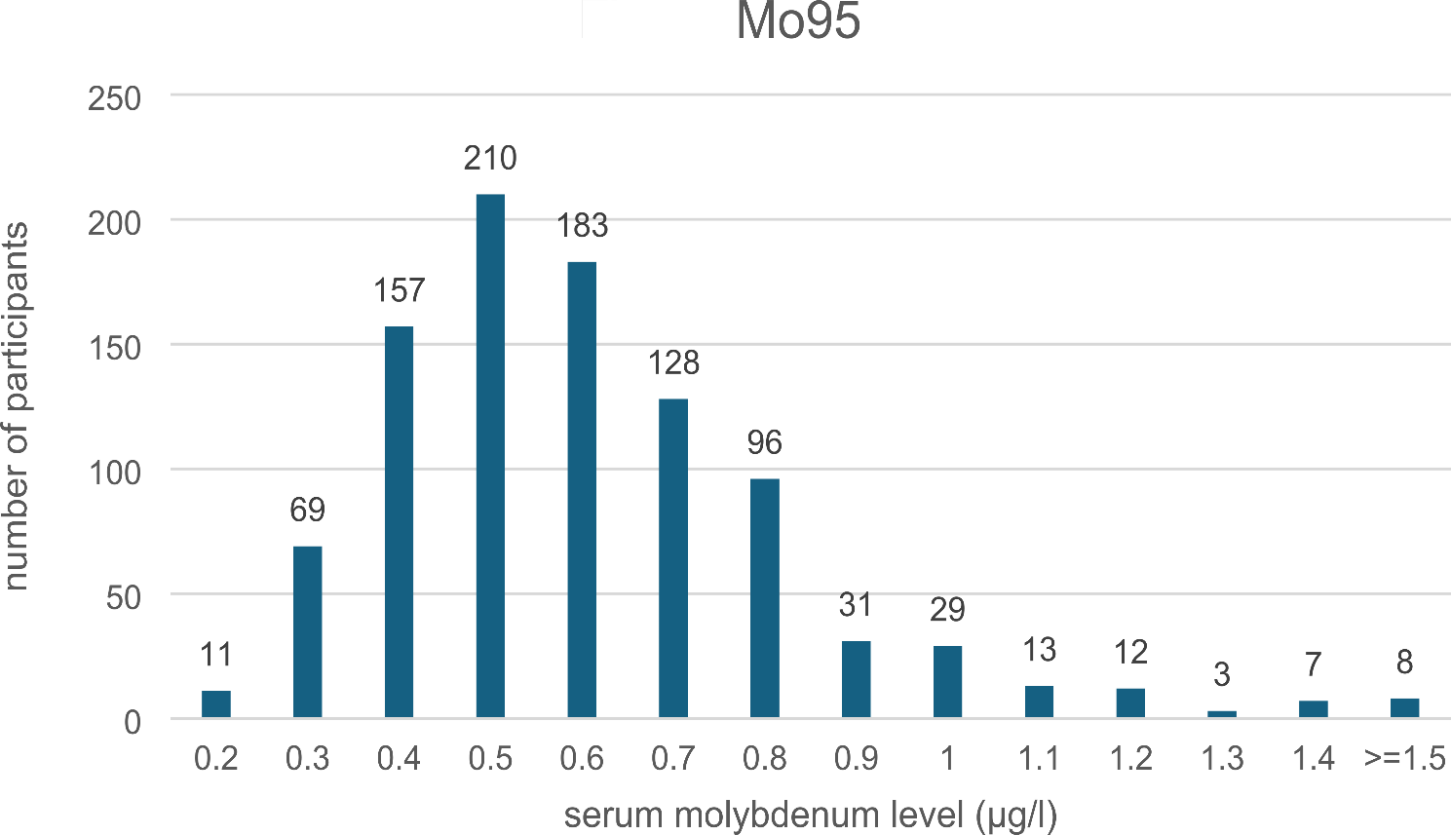
Illustrate the distribution of molybdenum levels in the study cohort

The 989 participants were assigned to one of three categories based on blood molybdenum levels. The distribution of molybdenum levels is shown in Fig. 1.

### Breast cancer

Women with blood molybdenum level above 0.7 µg/l had a modest 1.5-fold increased risk of developing breast cancer compared to women with levels below 0.54 µg/l (tertile 1 vs. 3; HR = 1.46; 95% CI:0.92–2.38) but this difference was not significant (*p* = 0.10) (Table 2).

**Table 2:** Hazard ratios for breast cancer according to molybdenum level

Variables	Breast cases/total	Univariate HR (95%CI) *P*-value	Multivariate* HR (95%CI) *P*-value
**Molybdenum level** (µg/l)			
≤ 0.53 0.53–0.70 > 0.70	30/317 36/319 48/321	1 1.12 (0.69–1.82) 0.65 1.46 (0.92–2.30) 0.11	1 1.13 (0.69–1.85) 0.63 1.46 (0.91–2.33) 0.12
**Year of birth**			
≤ 1965 1965.01–1975 1975.01–1985 > 1985	34/230 25/215 43/330 12/182	1 0.81 (0.48–1.35) 0.42 0.95 (0.61–1.50) 0.83 0.56 (0.29–1.08) 0.09	1 0.81 (0.31–2.11) 0.67 1.01 (0.28–3.72 )0.98 0.56 (0.14–2.28) 0.41
**Age at blood draw** (years)			
≤ 40 40.01-50 > 50	59/549 26/208 29/200	1 1.12 (0.71–1.78) 0.62 1.24 (0.79–1.94) 0.34	1 1.66 (0.65–4.25) 0.29 1.67 (0.45–6.24) 0.45
**Oophorectomy**			
No Yes	27/393 87/564	1 0.83 (0.57–1.21) 0.33	1 0.61 (0.37-1.00) 0.05
**Oral contraceptive use**			
No Yes	53/485 61/471	1 1.17 (0.81–1.69) 0.40	1 1.28 (0.86–1.90) 0.23
**Hormone replacement therapy**			
No Yes	83/697 31/259	1 0.87 (0.58–1.32) 0.51	1 0.86 (0.54–1.35) 0.50
**Smoking history**			
Never Current Former	54/539 33/215 27/203	1 1.60 (1.04–2.47) 0.03 1.33 (0.84–2.12) 0.22	1 1.62 (1.05–2.51) 0.03 1.31 (0.82–2.10) 0.26
**BMI at date of blood draw**			
≤ 23.0 > 23.0	57/450 54/470	1 0.88 (0.60–1.27) 0.49	1 0.85 (0.57–1.27) 0.43

* adjusted by all the variables listed in the left column

### Ovarian cancer

Initially unaffected women with blood molybdenum levels higher than 0.7 µg/l had a 5.5-fold greater risk of developing ovarian cancer compared to women with levels below 0.54 µg/l (tertile 3 vs. tertile 1, HR = 5.55; 95% CI: 1.59–19.4; *p* = 0.007). (Table 3).

**Table 3:** Hazard ratios for ovarian cancer according to molybdenum level

Variables	Ovarian cases/total	Univariate HR (95%CI) *P*	Multivariate* HR (95%CI) *P*
**Molybdenum level** (µg/l)			
≤ 0.53 0.53–0.69 > 0.69 Total	3/250 6/251 17/253 26/754	1 1.94 (0.49–7.78) 0.35 5.36 (1.51–18.3) 0.007	1 2.34 (0.57–9.64) 0.24 5.55 (1.59–19.4) 0.007
**Year of birth**			
≤ 1965 1965.01–1975 1975.01–1985 > 1985	9/95 8/156 8/321 1/182	1 0.50 (0.19–1.30) 0.15 0.25 (0.10–0.64) 0.004 0.06 (0.01–0.50) 0.009	1 1.08 (0.03–38.2) 0.97 0.34 (0.01–15.6) 0.58 0.08 (0.00-5.7) 0.25
**Age at blood draw** (years)			
≤ 40 40.01-50 > 50	12/539 5/124 9/91	1 1.81 (0.64–5.13) 0.27 4.70 (1.98–11.2) 0.0004	1 1.57 (0.13–2.42) 0.44 1.21 (0.03–53.8) 0.92
**Oral contraceptive use**			
No Yes	16/359 10/394	1 0.54 (0.25–1.20) 0.13	1 0.92 (0.38–2.24) 0.86
**Hormone Replacement Therapy**			
No Yes	23/601 3/152	1 0.44 (0.13–1.48) 0.19	1 0.35 (0.10–1.20) 0.10
**Smoking history**			
Never Current Former	11/436 6/170 9/148	1 1.40 (0.52–3.79) 0.51 2.53 (1.05–6.12) 0.04	1 1.38 (0.50–3.80) 0.53 2.40 (0.96–5.97) 0.06
**BMI at date of blood draw** (kg/m2)			
≤ 23.0 > 23.0	10/385 15/333	1 1.74 (0.78–3.87 )0.18	1 1.09 (0.44–2.68) 0.55

* adjusted by all the variables listed in the left column

The cumulative incidence of ovarian cancer at ten years was 1.2% for those in the lowest tertile, 4.2% for those in the middle tertile and 8.7% for those in the highest tertile. Fifty nine of the 754 women had a molybdenum level above 1.0 µg/l. For them, the ten-year cumulative incidence rate of ovarian cancer was 6.4%.

**Other cancers**: Overall, women in the highest tertile of molybdenum blood level (> 0.70) had a 1.7-fold increased risk of developing any cancer, in comparison to the lowest tertile of blood molybdenum (reference group) (adjusted HR = 1.74; 95% CI:1.17–2.61; *p* = 0.007) (data not shown).

## Discussion

In this prospective study of 989 *BRCA1* mutation carriers we observed a strong association between blood molybdenum level and the risk of ovarian cancer. In the unadjusted analysis, women in the highest tertile of blood molybdenum level (> 0.70 µg/L) had a 5.36-fold increased risk of developing ovarian cancer, compared to women in the reference group (*p* = 0.007). The hazard ratio was not modified by adjusting for main confounders (HR = 5.55; *p* = 0.007).

There was a moderate (not statistically significant) association between blood molybdenum and the incidence of breast cancers (adjusted HR = 1.46; *p* = 0.12).

A germline mutation in the BRCA1 gene predisposes individuals to the development of ovarian cancer, with the mechanism of loss of heterozygosity (LOH) playing a key role in this process [6]. The primary model for cancer development linked to BRCA1 mutations is based on the “two-hit” hypothesis, where one allele is lost, and the other undergoes a loss-of-function mutation [7].

Genomic instability arises from deficiencies in DNA repair caused by BRCA1 mutations, particularly affecting homologous recombination, leading to the accumulation of DNA damage and an increased risk of oncogenic mutations [8].

Additionally, oxidative stress plays a significant role in the pathogenesis of BRCA1-related cancers. BRCA1 indirectly influences oxidative stress regulation through the p53 protein, which responds to ROS-induced oxidative stress. BRCA1 is also essential for maintaining cell cycle checkpoints, particularly in response to DNA damage. Acting as an E3 ubiquitin ligase, BRCA1 polyubiquitinates G2/M cell cycle proteins, marking them for degradation and preventing their accumulation [9]. Mutations in BRCA1 allow cells with DNA damage to continue dividing, thereby increasing the risk of cancer development.

Exposure to certain heavy metals, such as lead [10] or molybdenum, has been identified as a potential environmental factor that could further exacerbate the risk of ovarian cancer, including individuals with BRCA1 mutations [11].

Molybdenum is an essential nutrient; the dietary requirement for adults is 45 µg/day (0.64 µg/kg/day) [12]. Exposure to excessive levels is associated with adverse health effects. The human body requires relatively small amounts of molybdenum, and it is provided through a normal diet. Molybdenum occurs naturally in a variety of foods, especially seeds, nuts, grains, beans, milk, offal and leafy vegetables [13]. Total molybdenum levels in the soil (and the plants growing therein) vary widely depending on geological composition and industrial contamination.

The Department of Health and Welfare and The Environmental Protection Agency (EPA) have not evaluated the potential carcinogenic effects of molybdenum in humans. Due to strict workplace standards, occupational poisoning is very rare. The source of chronic poisoning is most often inappropriate supplementation, less frequently a diet rich in foods with high molybdenum content (including crops grown on land enriched with fertilizers fortified with molybdenum chelates). Elevated levels in the air, water, and soil may be observed in proximity to industrial activities owing to contamination. Air pollutants mainly from coal mine combustion fall with rain and accumulate in the soil [14]. Poland has the highest levels of molybdenum in coal mine waste (2,332,000 µg/L) [15]. These levels far exceed the U.S. EPA health-based screening level of 40 µg/L [16]. There are varying sources of exposure to molybdenum - oral route 82%, inhalation 9% and dermal 9%.

Most of the work on the effects of molybdenum tumorigenesis dates back to the 1990s. At that time, a relationship between micronutrient levels and cancer risk was noted in animals.

Seventy-one animal and 24 human studies on molybdenum toxicity were found (including studies that found no effects). Of these, three were concerned with human cancers. Choi et al. [17] found significantly higher blood molybdenum levels in 150 Korean breast cancer patients, compared to 137 controls (1.16 µg/L vs. 1.05 µg/L; *p* = 0.004). It is worth noting the differences between our and Korean study. First of all, our study was prospective, whereas the Korean study was a case-control study. In addition, the Korean center studied serum, while we tested whole blood, these values are different, therefore it is not possible to compare them with the one obtained in our cohort.

A prospective study (*n* = 2,993) of metals and breast cancer risk in women without occupational exposure [18] analyzed the levels of 15 metals in toenails. They showed that higher molybdenum levels were associated with decreased breast cancer risk (HR = 0.82, CI = 0.76,1.00, *p* = 0.03). Our results differ possibly because the tissues studied were different and the level of heavy metals in the blood is closer to that which goes to internal organs such as the breast or ovary. In a case-control study of stainless-steel processing plant workers with molybdenum exposure an increased risk of lung cancer was shown (OR 2.1; 95% CI 1.2–3.7) [4].

Thus, the current information is insufficient to infer definite effects of exposure in humans. However, the data in our study may have important clinical implications. *BRCA1* carriers with high molybdenum levels should be encouraged to consider preventive oophorectomy. However, at present measuring blood micro-elements is not part of the standard clinical work up of *BRCA1* carriers and our results require confirmation. Blood molybdenum level can be also an attractive target for clinical trials aimed to decrease ovarian cancer risk on *BRCA1* carriers. The possibility of molybdenum’s potential toxicity and increased risk of tumorigenesis requires additional research.

## Conclusions

Blood molybdenum level is a maker of ovarian cancer risk on *BRCA1* mutation carriers. If confirmed, this association suggests that controlling blood molybdenum level through diet might reduce the risks of ovarian cancer in *BRCA1* positive women. Moreover, women with a *BRCA1* mutation and a high molybdenum level should be considered candidates for preventive oophorectomy.

## Patents

Based on the results presented in the following paper, a patent application has been sub-mitted to the Patent Office of the Republic of Poland (application ID P.447789).

## Data Availability

No datasets were generated or analysed during the current study.
